# A prospective observational study to evaluate the safety of COVID-19 mRNA vaccines administered to Qatar Rehabilitation Institute patients

**DOI:** 10.5339/qmj.2023.10

**Published:** 2023-03-01

**Authors:** Zahra Noureddine, Lama Madi, Sami Ullah, Haneen Alrawashdeh, Lina Naseralallah

**Affiliations:** ^1^Clinical Pharmacy Department, Qatar Rehabilitation Institute, Hamad Medical Corporation, Doha, Qatar. E-mail: ZNoureddine@hamad.qa ORCID: 0000-0001-9695-7293; ^2^Department of Physical Medicine and Rehabilitation, Qatar Rehabilitation Institute, Hamad Medical Corporation, Doha, Qatar; ^3^School of Pharmacy, College of Medical and Dental Sciences, University of Birmingham, Birmingham, UK

**Keywords:** COVID-19, adverse drug reactions, vaccine, mRNA, rehabilitation

## Abstract

Background: The safety of the COVID-19 mRNA vaccine in the outpatient setting has been extensively studied; however, there need to be more reports that specifically assess their safety in the inpatient population. It is hence imperative to explore the adverse drug reaction (ADR) profile in this population and monitor the progression of these ADRs in a hospital setting. This provides a unique opportunity to closely observe patients to ensure no side effects go undiagnosed. This study aims to explore and quantify the incidence and severity of ADRs in patients who have received the COVID-19 vaccine during their stay in the rehabilitation facility.

Methods: This is a prospective observational study, which included adult patients admitted to the rehabilitation facility who were deemed eligible to receive the COVID-19 vaccine during their hospital stay. Data were collected by the investigators from June 2021 to May 2022 at 24 hours, 48 hours, and 7 days post-vaccination. A piloted data collection tool was utilized.

Results: Thirty-five patients met the inclusion criteria. Pain at the injection site was the most commonly reported local ADR, while headache was the most frequent systemic ADR. The majority of the reported ADRs were mild to moderate in nature, with only one severe reaction detected. Although no statistical significance was noted among the variables, common patterns were identified, such as a higher occurrence of fever at 24 hours after the second dose as opposed to the first dose. Close monitoring of the included study subjects did not reveal any unanticipated ADRs or an increase in ADRs susceptibility and severity compared to the general population.

Conclusion: This study supports the initiation of vaccination campaigns in inpatient rehabilitation settings. This approach would offer the advantage of gaining full immunity and reducing the risk of contracting COVID-19 infection and complications once discharged.

## Introduction

Since the first case of coronavirus disease 2019 (COVID-19) was reported in Wuhan, China, in December 2019, this illness has spread to millions of people.^1^ So far, COVID-19 has resulted in more than 6 million deaths worldwide and over 500 million confirmed infections.^2^ An increased risk of severe disease and death has been noted among the elderly and people with preexisting medical conditions.^1^

Although several potential therapies appear to have produced promising results, to date, uncertainty still exists with regard to the exact therapeutic modalities.^3,4^ Vaccination represents an effective measure to prevent the viral spread and contain infectious diseases.^5^ During the years 2020–2021, clinically available COVID-19 vaccines have been developed at an unprecedented speed.^6^ According to the latest data from the World Health Organization, at least 10 different COVID-19 vaccines have been developed. The Food and Drug Administration has granted approval to only two messenger RNA (mRNA) COVID-19 vaccines: Pfizer-BioNTech and Moderna.^6^ mRNA technology works by triggering the immune cells to produce viral antigens in response to the administered mRNA fragments. Once the viral antigens are produced by the host cell, the normal adaptive immune response is activated. This eventually leads to the production of antibodies that are specifically targeted to the administered antigen, resulting in immunity.^7,8^

COVID-19 vaccines were developed and utilized rapidly, and as a result, their safety requires continuous surveillance. The safety of COVID-19 mRNA vaccines has been evaluated in multiple randomized clinical and observational studies. The published clinical trials assessed the safety of the vaccine in outpatient settings.^5,9^ In the outpatient cohort, pain, erythema, and swelling at the injection site were the most common local reactions, while headache, fatigue, myalgia, and nausea were the most common systemic adverse drug reactions (ADRs).^10–17^ While the majority of reported ADRs were mild to moderate in nature, severe ADRs have also been reported such as myocarditis and pericarditis.^18,19^

Investigating the safety of the vaccine in different clinical settings is yet to be explored. To our knowledge, there are no published studies that specifically assess the safety of COVID-19 mRNA vaccines in the inpatient population. Hence, it is imperative to explore the ADR profile in this population and monitor the progression of these ADRs in a hospital setting. The current study also provides a unique opportunity to closely observe patients to ensure no side effects go undiagnosed. The aim of this prospective observational study is to explore and quantify the incidence of ADRs following the administration of the mRNA vaccine in the inpatient population at the Qatar Rehabilitation Institute (QRI). The study also aims to explore the severity of the reported ADRs. QRI is a rehabilitation facility which offers support and rehabilitation to general trauma patients, stroke patients, as well as those suffering from spinal cord and traumatic brain injuries. Due to their prolonged hospital stay, these inpatients are candidates to receive the COVID-19 vaccine, which would enable clinicians to closely monitor any potential reactions to the vaccine.

## Methods

The reporting of the current observational study followed the STROBE (The Strengthening the Reporting of Observational Studies in Epidemiology) guideline.

### Study design and settings

A prospective observational study was conducted in QRI. QRI is the region's largest tertiary rehabilitation hospital managed by Hamad Medical Corporation (HMC); the principal public healthcare provider in the State of Qatar. QRI has approximately 193 beds, providing world-class integrated rehabilitation services to patients with a variety of neurological and physical disabilities. The length of stay for such patients is usually longer than in other facilities within HMC, as patients require intense physical and mental rehabilitation to be reintegrated back into the community. For this reason, those inpatients are candidates to receive the COVID-19 vaccine during their prolonged stay in QRI.

### Study population

All adult patients admitted to QRI who received the COVID-19 mRNA vaccine during the study period (June 2021 to May 2022) and who consented to enroll in the study were included. Although the institutional review board (IRB) granted the study 1 year, the research team collectively decided to stop recruitment after 5 months. This was due to the majority of the admitted patients being fully vaccinated at the time of admission. Therefore, it was expected that extending the recruitment period would not yield a significant additional number of study participants.

Patients who were not deemed eligible to receive the COVID-19 mRNA vaccine were excluded from this study. Patient exclusion criteria included the following:^20^

1. History of a severe allergic reaction (e.g., anaphylaxis) after a previous dose or to a component of the COVID-19 vaccine.
2. History of a known diagnosed allergy to a component of the COVID-19 vaccine.
3. Moderate or severe acute illness with or without fever (vaccination was deferred).
4. History of Multisystem Inflammatory Syndrome (MIS).
5. Patients who contracted COVID-19 within the last 6 months.

### Objectives

The primary objectives of this study were to explore and quantify the rate of ADRs in QRI inpatients receiving the COVID-19 mRNA vaccine and to explore the severity of the reported ADRs.

The secondary objectives were to identify any correlation between susceptibility and severity of ADRs after the first vs. second dose of vaccine, to identify any correlation between patient demographics and susceptibility to ADRs from the COVID-19 vaccine, and to identify any correlation between patient demographics and severity of ADRs (mild, moderate, or severe) from COVID-19 vaccine.

### Study procedures

The investigator obtained patient consent to be enrolled in the study. The study was explained to the patients and any inquiries or hesitancies were addressed by a member of the research team. Once consent was obtained, patients were asked to report their ADRs (if any). For feasibility reasons, ADRs were collected at three-time points; 24 hours, 48 hours, and 7 days post-vaccination.

ADR monitoring involved the following steps:

6. Identifying the ADRs.
7. Documentation of ADRs in patients’ medical records.
8. Reporting serious ADRs to pharmacovigilance centers/ADR regulating authorities.

### Data extraction and synthesis

The interview questions were guided by the data collection tool, which was created for the purpose of ADR monitoring/reporting. This tool was adopted and modified by Polack et al. and subsequently piloted with three patients.^10^ The severity of ADRs was categorized as mild, moderate, severe, or grade 4. Patients’ identification information was coded to maintain confidentiality.

### Sample size

The sample size was computed based on the primary outcome from the literature, and since there were no published studies which specifically included inpatients, a sample size was not calculated.

### Statistical analysis

Descriptive statistics were used to summarize demographics and other related parameters. All variables (ADR/No ADR, use of immunosuppressant medications, use of blood thinning medications, allergy, and gender) were expressed as numbers and percentages.

Association between two or more qualitative variables was assessed using the Chi-squared test and/or Fisher Exact test as appropriate. All *p*-values presented were two-tailed, and *p*-values < 0.05 were considered statistically significant. All statistical analysis was done using the statistical package SPSS 27.

## Results

### Characteristics of the study patients

During the study period, a total of 35 patients met the inclusion criteria and agreed to participate in the study. A summary of patients’ baseline characteristics is presented in Table 1. The majority of patients were male (85.7%), with their ages ranging between 30 and 60 years old (91.4%). Around half of the patients had a history of diabetes, hypertension, or stroke. Out of the 35 included patients, the use of blood thinning medication was confirmed in 29 (82.9%) patients. It is worth noting that all patients received the Pfizer-BioNTech mRNA vaccine.

### ADRs reported at 24 hours post-vaccination

The predominant local ADR was pain at the injection site (60%), of which moderate and severe reactions were reported in 11.4% and 2.9% of the patients, respectively (Table 2). Additionally, no significant reporting of other explored ADRs (redness and swelling) was noted.

Among systemic ADRs, the headache was the most common (17.1%), followed by fatigue (8.6%), fever (2.9%), chills (2.9%), and diarrhea (2.9%). Other reactions were documented in three patients (8.6%), which included nausea and dizziness. In terms of severity, all reported ADRs, except for two moderate headache incidents (5.7%), were mild. Six patients (17.1%) required antipyretic/analgesic within 24 hours post-vaccination.

### ADRs reported at 48 hours post-vaccination

Most local and systemic ADRs resolved or reduced in intensity after 48 hours (Table 3). Among local ADRs, pain at the injection site was the only identified reaction, with a 37% reduction as compared to the 24-hour follow-up point.

In total, there were only four systemic ADRs, of which two were headaches (one mild and one moderate), one was fatigue, and the other was chills. The need for antipyretic/analgesic medications decreased at 48 hours to 5.7% of the total included patients.

### ADRs reported at 7 days post-vaccination

Only 34 patients were included in the analysis at this time point, as one of the patients was discharged before completing the 7 days. All local and systemic ADRs resolved 7 days post-vaccination (Table 4), except for two cases of moderate headache. Antipyretic/analgesic consumption was also limited to two cases only.

### Relation between vaccine dose and the susceptibility and severity of ADRs

Fisher's exact test was utilized instead of Pearson's Chi-squared test whenever a number (i.e., cell) had an expected count less than five. Although no statistical significance was found, which could be due to the small sample size, common patterns were identified.

At the 24-hour follow-up session, only severe ADR (severe pain at the injection site) appeared after the first dose. Fever and the need for antipyretic/analgesics were more frequent after the second dose.

At the 48-hour follow-up session, mild pain at the injection site was more persistent after the first dose as compared to the second dose. In our patient cohort, all systemic ADRs presented only after the first dose of the COVID-19 vaccine. This means that even though the initial insignificant increase was observed in the number of febrile patients after the second dose, this fever was reduced after 48 hours.

At the 7-day follow-up session, no patterns (i.e., no notable increase or decrease) were identified.

### Relation between patient demographics and the susceptibility and severity of ADRs

The same approach followed to evaluate the correlation between the dose number and ADRs was employed in this section. No meaningful statistically significant difference was noted.

There was no elevated risk of either local or systemic ADRs observed in elderly patients, obese patients, and patients with a history of allergies at any of three-time points. Similarly, none of the explored comorbidities or concomitant medications led to an increase in the incidence or severity of ADRs.

## Discussion

The findings from the study demonstrated that pain at the injection site was the most common local ADR reported at 24 hours post-vaccination. Among systemic ADRs, the headache was the most frequent. The majority of local and systemic ADRs resolved or reduced in intensity after 48 hours of vaccination. By day 7, only two cases of moderate headache were reported; with the remainder of symptoms fully resolved. Only one severe ADR (pain at the injection site) was reported throughout the study, which partially improved at 48 hours and was fully resolved at 7 days. No unexpected ADRs were reported during the monitoring period. The statistical analysis showed no significance among the variables; however, common patterns were identified. For instance, there was a higher occurrence of fever at 24 hours after the second dose as opposed to the first dose.

Although this study's patient cohort was not previously investigated in the literature; the most common local ADR reported was consistent with previous studies conducted in diverse cohorts.

These studies consistently showed that local ADRs (such as pain at the injection site) and systemic ADRs (such as headache and fatigue) were the most reported after vaccination.^5,21,22^ A study conducted in the outpatient setting similarly revealed that injection site pain, headache, fever, and fatigue were among the most reported ADRs: 63.8%, 18.1 %, 4.3 %, and 3.2 %, respectively. The latter study also revealed the reporting of bone and muscle symptoms (7.4%), such as body aches and joint pain, which were not detected in this study.^23^ As the patients included in our study were recovering from events such as polytrauma, motor vehicle collisions, and strokes, there was the possibility of the existing pain masking the development of new musculoskeletal pain. Similarly, findings from a systematic review of three randomized controlled trials showed that local pain such as injection site reaction was the most commonly identified local ADR. However, in contrast to our findings, this review showed that these ADRs are more common in younger patients after the first dose, while our findings did not show any such pattern.^24^ Another review revealed that the most common local cutaneous ADRs were pain, erythema, and swelling.^25^ It is noteworthy that the current study did not show any significant occurrence of erythema or swelling at the injection site.

In terms of frequency of ADRs, the Centers for Disease Control and Prevention (CDC) reported fatigue as the most common systemic ADR after the mRNA vaccine, followed by headache.^26^ In this study, the headache was the most common systemic ADR, followed by fatigue. This finding should be interpreted in the context of the special characteristics of included patients. The rehabilitation institute admits patients who require neurological and physical rehabilitation with risk factors that potentially predispose them to experience headaches.^27^ The reported headaches were transient and mild-to-moderate in nature, and their onset was within the first 24 hours (17.1%). They were not associated with any systemic illness; hence, they did not interfere with the patients’ rehabilitation schedule. This aligns with findings from a retrospective review of data collected from the United States-based Vaccine Adverse Reporting System, which showed that different COVID-19 vaccines were associated with headaches mostly experienced within 24 hours of vaccination and such headaches were not linked with systemic illness such as cerebrovascular events.^28^ This finding is also consistent with findings from a systematic review of three randomized trials where fatigue, headache, and myalgia were the most commonly reported systemic ADRs.^24^

The incidence of fever in our population group was higher following the second dose administration. Although this pattern was not statistically significant, this finding was in line with the reported data from the literature.^5,9,10,26^ This could be explained by the immune system's response mechanism. The first dose activates the immune system; however, the full immune response is only achieved after administering the second dose, hence explaining the higher incidence of fever.^29^ A systematic review and meta-analysis of 13 randomized controlled trials also showed that fever, fatigue, and bodily pain were the most common severe ADRs.^30^

In the literature, severe adverse events like hypersensitivity, facioplegia, urticaria, and anaphylactic shock were rare; none of them were detected in the current study.^31^ Similarly, the CDC reported rare occurrences of the following severe systemic ADRs: anaphylaxis, thrombosis, and myocarditis/pericarditis.^32^ The CDC also reported that myocarditis/pericarditis was more common with mRNA vaccines as opposed to other vaccines (2 to 32 cases per 100,000 person years).^33^ A systematic review of case reports and case series showed that among cardiac ADRs, myocarditis/myopericarditis and pericarditis were the most common.^34^ No cases of myocarditis/pericarditis were detected in the study's subjects. Furthermore, according to the reports submitted to the Pharmacovigilance Systems in the European Union and the United States, the risk estimate of thrombotic events was 3.31 per 100,000 exposed individuals.^35^ Due to the small sample size of this study, none of the aforementioned severe ADRs were detected; hence, a larger sample size would need to be recruited to allow for the detection of such events.

To our knowledge, this is the first prospective study to evaluate and assess the safety of COVID-19 mRNA vaccines in hospitalized patients. Due to their prolonged hospital stay, vaccinating this cohort of patients offers the advantage of gaining full immunity by the time of discharge and ensuring patients are protected against COVID-19 in the community. Additionally, hospital inpatients are at risk of missing out on the opportunity to receive timely vaccine doses; hence, offering inpatient vaccination helps eliminate this risk.^36^ As the results of this study did not show an increased risk of COVID-19 vaccine ADRs in the inpatient population, administering the vaccine during the patient's hospital stay is highly supported.^36–39^ This is pivotal, as this patient cohort has been proven to be at a higher risk for COVID-19 complications if the virus was to be contracted.^40,41^ The setting allowed for the close observation and monitoring of patients after receiving both doses of the COVID-19 vaccine. The research team's in-depth conversations with the patients did not reveal any additional or unanticipated side effects. Furthermore, the small size of the research team offered the advantage of having a standardized approach to data collection.

This study had several limitations that should be acknowledged. The main limitation of this study was the small sample size; thus, less common ADRs were not detected. The intention was to recruit the highest number of patients during the IRB approval duration; however, due to delays in obtaining the approval, the majority of the population had already been vaccinated. Hence, fewer patients met our inclusion criteria, resulting in a small sample size. Additionally, the 7-day follow-up period did not provide sufficient time for the development and detection of delayed ADRs. Lastly, due to feasibility reasons, the study site was limited to one rehabilitation hospital.

The patient cohort included in this study coincidently received the Pfizer-BioNTech mRNA vaccine. Previous reports conducted in outpatient settings showed that both brands of mRNA vaccine (Pfizer-BioNTech and Moderna) had similar ADR profiles; however, the frequency was found to be lower with the Pfizer-BioNTech vaccine.^42^ Future studies could compare the safety of both mRNA vaccines in the inpatient rehabilitation population to confirm these results. Additionally, future work could explore and assess the safety of COVID-19 vaccines on a larger cohort of patients, while expanding the study site to include other rehabilitation facilities.

## Conclusion

The results of this study did not show an increased risk of ADRs following vaccination with the Pfizer-BioNTech mRNA vaccine in the inpatient population. The findings from this study closely aligned with the published literature on outpatients, providing support for inpatient vaccination campaigns. Concerns about the interference of inpatient vaccination with the patient's rehabilitation schedule were not supported. As a result, delaying the vaccine until patients complete their rehabilitation is unjustifiable. It is hence recommended to initiate vaccination campaigns in inpatient facilities, while the patients are completing their rehabilitation program.

### Conflicts of interest

All authors declare that they have no conflict of interest.

### Ethical statement

The study was approved by the Medical Research Center, Hamad Medical Corporation (HMC), in June 2021 (MRC-01-21-059). HMC-IRB Registration: IRB-HMC-2021-011. IRB-MoPH Assurance: IRB-A-HMC-2019-0014.

### Funding

The authors received no financial support for this research.

### List of abbreviations

ADRs: Adverse drug reactions

CDC: Centers for Disease Control and Prevention

COVID-19: Coronavirus disease 2019

FDA: Food and Drug Administration

GBS: Guillian–Barre Syndrome

HMC: Hamad Medical Corporation

IRB: Institutional Review Board

MIS: Multisystem inflammatory syndrome

mRNA: Messenger RNA

QRI: Qatar Rehabilitation Institute

STROBE: The Strengthening the Reporting of Observational Studies in Epidemiology

TTS: Thrombocytopenia syndrome

VAERS: Vaccine Adverse Reporting System

WHO: World Health Organization

## Figures and Tables

**Table 1:** The demographic characteristics of the study population.

Variable		Total: *n* = 35, no. (%)
Age (Years)	30–40	13 (37.1)
	41–50	11 (31.4)
	51–60	8 (22.9)
	>60	3 (8.6)
Gender	Male	30 (85.7)
	Female	5 (14.3)
Body mass index (BMI)	Underweight (BMI < 18.5)	0 (0)
	Normal weight (BMI 18.5–24.9)	19 (54.3)
	Overweight (BMI 25–29.9)	8 (22.9)
	Obese (BMI ≥ 30)	8 (22.9)
Allergies	No allergies	30 (85.7)
	Food allergy	2 (5.7)
	Drug allergy	3 (8.6)
Dose number	First dose	22 (62.9)
	Second dose	13 (37.1)
Comorbidities		
Diabetes	No	17 (48.6)
	Yes	18 (51.4)
Hypertension	No	17 (48.6)
	Yes	18 (51.4)
Stroke	No	18 (51.4)
	Yes	17 (48.6)
Coronary artery disease	No	29 (82.9)
	Yes	6 (17.1)
Other medications		
Immunosuppressants	No	33 (94.3)
	Yes	2 (5.7)
Blood-thinning medications	No	6 (17.1)
	Yes	29 (82.9)

**Table 2:** ADRs were reported at 24 hours post-vaccination.

Reported ADRs		Total: *n* = 35, no. (%)
Local ADRs		
Pain at the injection site	No	14 (40)
	Mild	16 (45.7)
	Moderate	4 (11.4)
	Severe	1 (2.9)
Redness	No	35 (100)
	Mild	0 (0)
	Moderate	0 (0)
	Severe	0 (0)
Swelling	No	34 (97.1)
	Mild	1 (2.9)
	Moderate	0 (0)
	Severe	0 (0)
Systemic ADRs		
Fever	No	34 (97.1)
	Mild	1 (2.9)
	Moderate	0 (0)
	Severe	0 (0)
Fatigue	No	32 (91.4)
	Mild	3 (8.6)
	Moderate	0 (0)
	Severe	0 (0)
Headache	No	29 (82.9)
	Mild	4 (11.4)
	Moderate	2 (5.7)
	Severe	0 (0)
Chills	No	34 (97.1)
	Mild	1 (2.9)
	Moderate	0 (0)
	Severe	0 (0)
Vomiting	No	35 (100)
	Mild	0 (0)
	Moderate	0 (0)
	Severe	0 (0)
Diarrhea	No	34 (97.1)
	Mild	1 (2.9)
	Moderate	0 (0)
	Severe	0 (0)
New or worsening muscle pain	No	35 (100)
	Mild	0 (0)
	Moderate	0 (0)
	Severe	0 (0)
New or worsening joint pain	No	35 (100)
	Mild	0 (0)
	Moderate	0 (0)
	Severe	0 (0)
Use of antipyretics	No	29 (82.9)
	Yes	6 (17.1)
Serious reaction	No	35 (100)
	Yes	0 (0)
Other reactions	No	32 (91.4)
	Yes	3 (8.6)

ADR: adverse drug reaction

**Table 3:** ADRs were reported at 48 hours post-vaccination.

Reported ADRs		Total: *n* = 35, no. (%)
Local ADRs		
Pain at the injection site	No	27 (77.1)
	Mild	8 (22.9)
	Moderate	0 (0)
	Severe	0 (0)
Redness	No	35 (100)
	Mild	0 (0)
	Moderate	0 (0)
	Severe	0 (0)
Swelling	No	35 (100)
	Mild	0 (0)
	Moderate	0 (0)
	Severe	0 (0)
Systemic ADRs		
Fever	No	35 (100)
	Mild	0 (0)
	Moderate	0 (0)
	Severe	0 (0)
Fatigue	No	34 (97.1)
	Mild	1 (2.9)
	Moderate	0 (0)
	Severe	0 (0)
Headache	No	33 (94.2)
	Mild	1 (2.9)
	Moderate	1 (2.9)
	Severe	0 (0)
Chills	No	34 (97.1)
	Mild	1 (2.9)
	Moderate	0 (0)
	Severe	0 (0)
Vomiting	No	35 (100)
	Mild	0 (0)
	Moderate	0 (0)
	Severe	0 (0)
Diarrhea	No	35 (100)
	Mild	0 (0)
	Moderate	0 (0)
	Severe	0 (0)
New or worsening muscle pain	No	35 (100)
	Mild	0 (0)
	Moderate	0 (0)
	Severe	0 (0)
New or worsening joint pain	No	35 (100)
	Mild	0 (0)
	Moderate	0 (0)
	Severe	0 (0)
Use of antipyretics	No	33 (94.3)
	Yes	2 (5.7)
Serious reaction	No	35 (100)
	Yes	0 (0)
Other reactions	No	35 (100)
	Yes	0 (0)

ADR: adverse drug reaction

**Table 4:** ADRs were reported at 7 days post-vaccination.

Reported ADRs		Total: *n* = 34, no. (%)
Local ADRs		
Pain at the injection site	No	34 (100)
	Mild	0 (0)
	Moderate	0 (0)
	Severe	0 (0)
Redness	No	34 (100)
	Mild	0 (0)
	Moderate	0 (0)
	Severe	0 (0)
Swelling	No	34 (100)
	Mild	0 (0)
	Moderate	0 (0)
	Severe	0 (0)
Systemic ADRs		
Fever	No	34 (100)
	Mild	0 (0)
	Moderate	0 (0)
	Severe	0 (0)
Fatigue	No	34 (100)
	Mild	0 (0)
	Moderate	0 (0)
	Severe	0 (0)
Headache	No	32 (94.1)
	Mild	0 (0)
	Moderate	2 (5.9)
	Severe	0 (0)
Chills	No	34 (100)
	Mild	0 (0)
	Moderate	0 (0)
	Severe	0 (0)
Vomiting	No	34 (100)
	Mild	0 (0)
	Moderate	0 (0)
	Severe	0 (0)
Diarrhea	No	34 (100)
	Mild	0 (0)
	Moderate	0 (0)
	Severe	0 (0)
New or worsening muscle pain	No	34 (100)
	Mild	0 (0)
	Moderate	0 (0)
	Severe	0 (0)
New or worsening joint pain	No	34 (100)
	Mild	0 (0)
	Moderate	0 (0)
	Severe	0 (0)
Use of antipyretics	No	32 (94.1)
	Yes	2 (5.9)
Serious reaction	No	35 (100)
	Yes	0 (0)
Other reactions	No	35 (100)
	Yes	0 (0)

ADR: adverse drug reaction
